# A Clinical and Ethical Dilemma: Expectant Management for Ectopic Pregnancy with a Vital Fetus in a Low-Resource Setting

**DOI:** 10.3390/jcm12175642

**Published:** 2023-08-30

**Authors:** Michele Orsi, Foday Musa Janneh, Amadu Sesay, Abdul Karim Bah, Nitsuh Addis Tiru

**Affiliations:** 1 Unit of Obstetrics, Department of Woman Newborn and Child, Fondazione Istituto di Ricovero e Cura a Carattere Scientifico Ca’ Granda Ospedale Maggiore Policlinico, Via della Commenda, 12, 20122 Milan, Italy; 2 Princess Christian Maternity Hospital, University of Sierra Leone Teaching Hospital Complex, Fourah Bay Road, Freetown 00232, Sierra Leone; 3 Doctors with Africa CUAMM, Via San Francesco, 126, 35121 Padova, Italy

**Keywords:** ectopic pregnancy, expectant management, female infertility, medical ethics, assisted reproduction, low-resource setting, Sierra Leone

## Abstract

Background: Guidelines recommend the prompt surgical removal of any ectopic pregnancy (EP) in the presence of a vital embryo. This treatment impacts future fertility, particularly in low-resource settings where access to assisted reproductive techniques is limited. In addition, growing evidence is reporting live births after conservative management of initially undiagnosed abdominal pregnancies. Therefore, the discussion on the acceptability of expectant management in selected cases has been recently raised. Case: We present and discuss the case of a woman with vital first trimester EP who refused surgical treatment at Princess Christian Maternity Hospital, Freetown, Sierra Leone. She was initially diagnosed with a 12 week pregnancy located in the left adnexal region without hemoperitoneum. She refused both surgical treatment and hospital admission and did not come back to the hospital for antenatal care until 26 weeks of gestational age. Therefore, she was admitted and finally delivered, at 34 weeks of gestation, a 1.9 kg healthy baby which was alive. To disentangle the potential conflict between the ethical principles of medical treatment’s beneficence and the patient’s autonomy, we provide an update on counselling for a patient with early vital EP in a resource-limited setting and discuss the knowledge gap in this area. Conclusions: Limited access to fertility treatment in low- and middle-income countries may justify the discussion of expectant management as an option in selected cases of uncomplicated vital EP.

## 1. Teaching Points

- Growing evidence reporting successful cases of abdominal pregnancy challenges the traditional assumption that ectopic pregnancy is non-viable by definition;
- The report provides an update regarding the potential conflict between the ethical principles of beneficence and the patient’s autonomy while managing a vital ectopic pregnancy;
- The limited access to infertility treatment in low- and middle-income countries suggests bringing forward the debate regarding expectant management in uncomplicated cases of ectopic pregnancy.

## 2. Background

Ectopic pregnancy (EP) accounts for 1–2% of all pregnancies and is the medical emergency responsible for the majority of maternal deaths occurring during the first trimester of gestation [1,2,3]. Most cases arise from the tubal implantation of the embryo [4]. Signs and symptoms of an imminent or ongoing rupture, hemodynamic instability or the presence of a viable embryo are absolute indications for prompt surgical intervention [5].

However, the woman may sometimes refuse the operation for a variety of reasons [6,7]. First of all, the surgical treatment mainly entails salpingectomy. This intervention does not entirely hamper the chances of a future pregnancy, but inevitably may evoke in women thoughts of castration. In fact, in cases of previous contralateral salpingectomy, it may result in a sterilizing intervention. These aspects should be extensively discussed during the preoperative counselling [8,9]. Moreover, it must be considered that many women suffering from EP have a history of infertility, since several factors underlie both conditions [10,11,12]. Therefore, the choice of terminating a viable pregnancy may sound conceptually unacceptable, even if it is a life-saving procedure [13]. In some cases, it may also entail relevant psychological consequences, including post-traumatic stress disorder, depression or suicide [14,15].

This situation raises an ethical dilemma for healthcare providers [6,13]. A conflict may arise between the principles of patient autonomy and beneficence. The patient’s wish to carry on the pregnancy collides with the healthcare providers’ treatment proposal to mitigate risk and improve survival [16]. In low-resourced settings, challenged with low educational levels, cultural, and religious peculiarities; a fragile healthcare system and limited access to optimal infertility treatment, this conflict becomes more distinct [17,18,19,20].

Guidelines [5] recommend surgical removal of vital EPs based on the general assumption that they cannot generate a live birth while posing significant risk to patients’ lives [13]. Nonetheless, growing evidence has shown favorable outcomes in cases of undiagnosed abdominal pregnancies (AP), some of which originated from tubal sites [21,22,23]. This has stimulated the discussion about expectant management of EP in selected cases [21,22].

Through the description of a challenging case with vital first-trimester EP, that refused surgery at the main teaching referral facility for obstetrics and gynecology in Freetown, Sierra Leone, we discuss some critical issues of concern in the shared decision-making process.

## 3. Case Presentation

**Figure 1:**
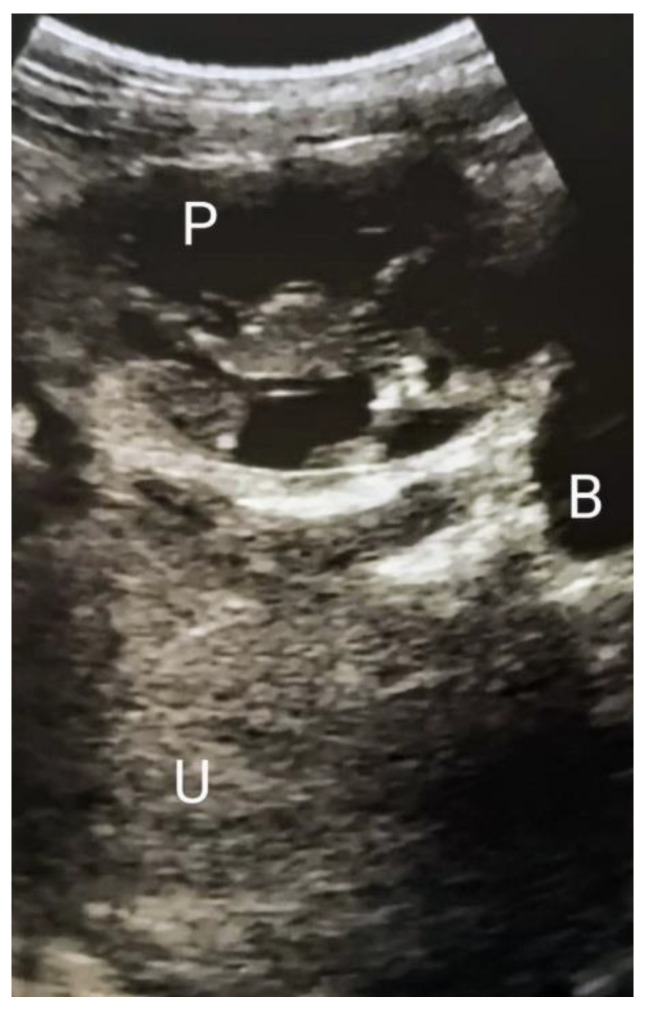
First trimester abdominal ultrasound scan showing the abdominal pregnancy, the empty uterus, and the absence of free fluid in the abdomino–pelvic cavity. P = pregnancy; U = uterus; B = bladder.

A 26-year-old gravida 2 para 0, was referred to Princess Christian Maternity Hospital, Freetown, Sierra Leone, with amenorrhea of three months duration a three day history of worsening pelvic pain. She had a prior history of right salpingectomy performed 3 years before presentation as a result of ruptured right tubal EP. On examination, there was moderate tachycardia and lower abdominal tenderness; the pregnancy test was positive, and the transabdominal ultrasound scan revealed a 12-weeks left adnexal EP with a vital fetus and no free fluid collection in the pouch of Douglas (Figure 1). In this public academic hospital No other hematological diagnostic or imaging investigations were available, and they were not included in the free healthcare guaranteed by the national program. Blood tests and magnetic resonance imaging are carried out in other private institutions. A rapid test for hemoglobin was performed and the result was within the normal range. In the presence of the husband, the patient was offered detailed counselling for the management of her condition. Considering the vital fetus, the global consensus indicates the surgical removal of the pregnancy. As a symptom, pain could indicate impending or initial tubal rupture, and the consequent bleeding potentially threatens the patient’s survival. Therefore, emergency exploratory laparotomy was proposed for suspicion of the imminent rupture of the EP.

**Figure 2:**
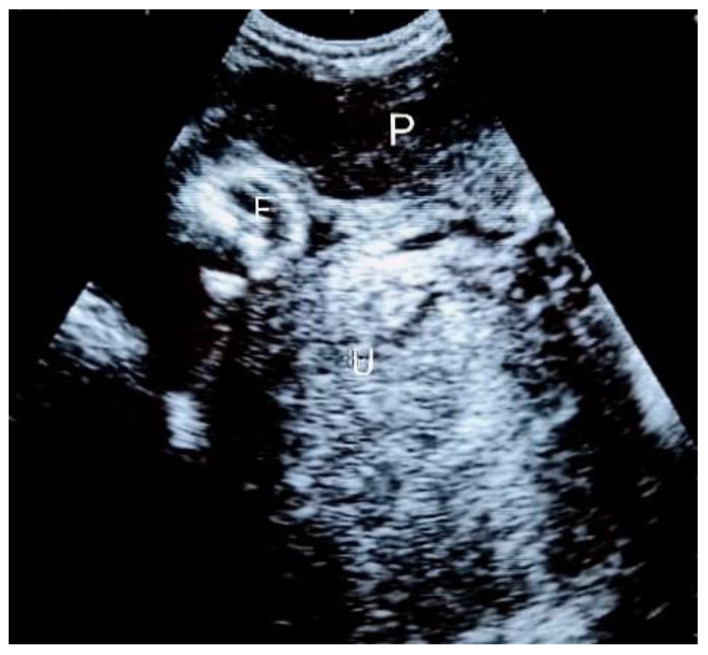
Third trimester abdominal ultrasound scan (left-lateral view) showing the fetal limbs, placenta, amniotic fluid, and the empty uterus. P = placenta; F = fetal limbs; U = uterus.

Considering the previous salpingectomy and subsequent fertility implications, the patient outrightly refused both surgery and hospital admission, even after the proposal to only be hospitalized and remain monitored without immediate intervention. Analgesics and hematinics were then prescribed and a short-term outpatient follow-up was then advised. She defaulted on antenatal follow-up visits, and she avoided responding to repeated attempts of the medical staff to contact her. She only came to the hospital at 26 weeks of gestation. An ultrasound scan was then repeated (Figure 2). The fetus was still vital and growing within the normal range, with adequate amniotic fluid and normal umbilical flow at the Doppler ultrasound assessment. She was then admitted for close monitoring and expectant management. Fetal lung maturation was induced by maternal intramuscular injection of corticosteroids at 28 weeks of gestational age, in order to reduce the risk of severe neonatal respiratory distress syndrome. 

Eventually, a healthy baby girl weighing 1.9 kg was delivered by laparotomy at 34 weeks of gestational age, with an APGAR score of 8 in one minute and 9 in five minutes, and was referred to the special care baby unit. No major anatomical abnormalities of the newborn were noted, with the exception of mild contractures of the lower limbs. The placenta was firmly adherent to the left lower abdomino–pelvic wall, the annexus, the broad ligament and the sigmoid colon on the same side. Therefore, a decision was made to provide essential hemostasis and leave it in situ in order to prevent a potentially dramatic hemorrhage. Total estimated blood loss was 1200 mL and 2 units of whole blood were transfused. Tranexamic acid and broad-spectrum antibiotics were therefore prescribed and 12 h after the surgery, when the patient was hemodynamically stable, anti-thrombotic prophylaxis with low molecular weight heparin was introduced. The mother and the baby recovered well and were discharged home 2 weeks later.

## 4. Discussion

The diagnosis of EP signifies a potentially life-threatening condition, and all possible efforts must be targeted at mitigating the related risks. Healthcare providers should offer such patients updated and evidence-based counselling for shared decision-making [24].

To the best of our knowledge, this is the first report discussing the potential role of conservative management of a first trimester ectopic gestation with a vital fetus. In our case, clinical and ethical challenges arose from the patient’s refusal of the surgical treatment. There are no guidelines that suggest the management of a situation of this type. However, this case raises questions about the current recommendations in light of the peculiarity of the context, with limited resources, and the clinical characteristics of the patient.

Guidelines are designed on the basis of robust evidence mostly derived from randomized trials, while the role of case reports is to present insights or controversies that can inspire subsequent investigations and appropriate studies.

In view of the large number of human lives lost due to complications of ectopic pregnancy, prompt management is recommended to protect women’s health [25]. Therefore, the discussion of expectant management is not currently an option, and in the event of a patient’s request, it must be evaluated with caution while taking into account the available diagnostic and therapeutic resources as well as the socio–cultural context [6]. Furthermore, considering the high likelihood of complications, it is a shared opinion that emphasizing the very low chance of an EP resulting in a live birth facilitates the convergence of the patient–physician dyad towards the choice to immediately treat this condition. However, we believe that providing updated and detailed counseling to the patient with EP is an absolute priority in order to guarantee the patient’s full decision-making autonomy [6]. In this regard, the now-no-longer anecdotal evidence of successful ectopic pregnancies should be cited, while emphasizing the risk of mortality and the high burden of maternal and fetal complications [21].

To support clinicians facing this condition and to deepen the ethical basis of the counselling while taking into account the recent evidence in this field, we focused the discussion on some typical questions that a patient diagnosed with EP may ask to the healthcare provider.

*“Considering that the pregnancy is progressing, is there any actual risk for my life?”*

The diagnosis of EP is itself a high-risk condition. In the presence of complications, such as hemoperitoneum or hemodynamic instability, there is an absolute indication for urgent surgical treatment [5]. This condition is potentially fatal and the risk increases in cases of failed diagnosis and delayed treatment [1,2,26]. Therefore, awareness of this condition and availability of prompt management are deemed to be protective factors. In the absence of hemoperitoneum, EP cannot be considered an immediate threat to the patient’s life, but it is still a very high-risk condition. In keeping with the ethical principle of beneficence and in accordance with international guidelines, surgical removal should be proposed in the case of a viable EP [5,16].

*“For my tubal pregnancy, is there any chance to success? In other words, what is the risk of rupture versus the chance to deliver a live baby?”*

The thickness of the tube’s mucosa and sub-mucosa are inadequate to sustain trophoblastic invasion and support the pregnancy progression. Therefore, most tubal EP are expected to rupture and cause intra-abdominal hemorrhage. However, after tubal abortion or rupture, trophoblasts may sometimes invade adjacent abdominal organs, such as omentum, peritoneum, and others, to acquire sufficient blood and nutrients supply and survive beyond the first trimester without causing life-threatening bleeding [27,28]. The result is an AP, whose incidence remains currently unknown because most EPs are treated in the first trimester. Secondary AP remains a major diagnostic challenge [21,22], with an intraoperative diagnosis rate of up to 50% in both high- and low-income countries [29]. Ultrasound features of AP include absence of the uterine wall between the maternal bladder and the fetus; ectopic location of placenta; abnormal presentation of fetus; proximity of fetal parts to maternal abdominal wall; and absence of amniotic fluid between the placenta and the fetus [30]. The diagnosis of secondary AP can be made when the following criteria for primary AP are not verified: (1) normal tubes and ovaries; (2) absence of uteroplacental fistula; and (3) sufficiently early diagnosis to exclude the possibility of secondary implantation [31,32].

Since reports of advanced undiagnosed AP with successful outcome have been published, some authors started promoting a conservative management in selected cases [7,21,22]. A recently published case series from a South African referral center reported the outcomes of 118 advanced abdominal pregnancies [21] over a 22-year study period. Of these cases, 51 underwent immediate delivery for a viable fetus, while 46 underwent expectant management. In this wait-and-see group, 2 cases (4%) resulted in stillbirth, 11 (24%) in perinatal death, and 33 (72%) women were discharged from the hospital with a live newborn. Out of 46 cases, there were no maternal deaths, but 39 patients received hemotransfusions, 1 required relaparotomy and 1 had an intestinal lesion successfully repaired intraoperatively. Finally, the reported risk of congenital anomalies and severe prematurity is significantly higher than for abdominal pregnancies in comparison to intrauterine pregnancies [21,22].

Therefore, these reports as long as the existing knowledge gaps should be disclosed during the counseling, to fully guarantee the ethical principle of the patient’s autonomy [16,24,33].

*“But I’m infertile. What if this was my only chance? What if I refuse the surgery?”*

In low- and middle-income countries, the vast majority of patients have limited access to assisted reproductive techniques (ART) for the treatment of infertility [34,35]. Therefore, the refusal of surgery in our case—in the patient who had previous contralateral salpingectomy—could be understandable from the fertility–preservation point of view. Although it is not possible to draw a full resemblance, if a pregnant woman with a pre-existing high-risk medical condition—such as pulmonary arterial hypertension or heart failure—refuses to terminate her pregnancy, the situation would compel clinicians to design a new personalized care pathway [36]. Although these conditions correlate with a risk of maternal death of up to over 30%, detailed and comprehensive counseling on pregnancy success and failure rates in cases described in the literature should be offered. An even closer comparison could be made with cesarean scar pregnancy, which in the case of wait-and-see management exposes the patient to a significant risk of hemorrhage, uterine rupture, premature birth, hysterectomy, and death [37].

If a patient with an EP opts for conservative management against medical advice, we recommend close monitoring, including hospitalization, if necessary. Since most maternal deaths from EP occur as a result of delayed diagnosis or treatment [2,26,38,39], the awareness of this condition is expected to play a protective role and minimize mortality. Although this information is not available in the literature, we reasonably assume that the risk of severe outcomes is dramatically reduced if cross-matched blood units are prepared, and an adequately experienced surgical team is available on call. We are aware that these conditions require a considerable expenditure of healthcare resources. However, they are theoretically available as well as a basic requirement for referral centers even in low-resource countries.

## 5. Conclusions

According to guidelines, a patient with a first trimester vital ectopic pregnancy should promptly undergo its surgical removal. However, no recommendations are available to manage cases that refuse treatment. Furthermore, growing evidence is reporting successful cases of live births from AP, some of which originate from the fallopian tube. This evidence challenges the traditional assumption that EP is non-viable by definition and discloses a relevant knowledge gap in this area. In this case, considering the frequent coexistence of subfertility among cases of EP, the impact of its surgical treatment, and the limited access to ART in LMIC, clinicians should bring forward the debate regarding expectant management in highly selected cases. Providing the patient with comprehensive counseling would ensure the ethical principle of the patient’s autonomy and foster the convergence with the principle of beneficence supported by the healthcare provider towards a shared decision-making process.

## Data Availability

The data created during this current study are available from the corresponding author on reasonable request.
